# Implementation and scientific evaluation of rehabilitative sports groups for prostate cancer patients: study protocol of the ProRehab Study

**DOI:** 10.1186/1471-2407-12-312

**Published:** 2012-07-24

**Authors:** Eva M Zopf, Moritz Braun, Stefan Machtens, Jürgen Zumbé, Wilhelm Bloch, Freerk T Baumann

**Affiliations:** 1 Institute of Cardiovascular Research and Sport Medicine, Department of Molecular and Cellular Sport Medicine, German Sport University Cologne, Am Sportpark Müngersdorf 6, Cologne, 50933, Germany; 2 Heilig-Geist-Hospital Cologne-Longerich, Graseggerstr. 105, Cologne-Longerich, 50737, Germany; 3 Marien Hospital Bergisch Gladbach, Dr.-Robert-Koch-Str. 18, Bergisch Gladbach, 51465, Germany; 4 Clinical Centre Leverkusen, Am Gesundheitspark 11, Leverkusen, 51375, Germany

**Keywords:** Prostate cancer, Rehabilitative sports groups, Physical activity, Exercise

## Abstract

**Background:**

Although treatment regimen have improved in the last few years, prostate cancer patients following a radical prostatectomy still experience severe disease- and treatment-related side effects, including urinary incontinence, erectile dysfunction and psychological issues. Despite high incidence rates and the common adverse effects there is a lack of supportive measures for male patients and specific physical exercise recommendations for prostate cancer patients during rehabilitation or in the aftercare are still missing.

**Methods/Design:**

The ProRehab Project aims to establish rehabilitative sports groups particularly for prostate cancer patients and to evaluate the effects of the offered exercise program. Starting 8–12 weeks after prostatectomy or combination therapy, prostate cancer patients will exercise for 15 months within a patient preference randomized controlled trial. One exercise session will be conducted within a pre-established rehabilitative sports group, while the other will be completed independently. Patients in the control group will not participate in the intervention. The main outcomes of the study include aerobic fitness, quality of life, incontinence and erectile dysfunction.

**Discussion:**

By combining science, practice, and public relations the first rehabilitative sports groups for prostate cancer patients in Germany have been set up and thus contribute to the care structure for prostate cancer patients. By offering a 15-month physical exercise intervention that is conducted in supervised group sessions, long-term lifestyle changes and therefore improvements in quality of life in prostate cancer patients can be expected.

**Trial registration:**

German Clinical Trials Register DRKS00004184

## Background

Despite improved treatment regimes and diagnostic methods, the consequences of a prostate cancer (PCa) disease and its medical treatment are profound. The most common side effects caused by radical prostatectomy or irradiation are urinary incontinence and erectile dysfunction [1,2]. Depending on the surgical technique, the surgeons experience, the definition of urinary incontinence, and the method of assessment, 5-74% PCa patients experience urinary incontinence after prostate resection [3,4]. While most patients will recover at least partially, others will have to live with an irreversible incontinence. The incidences of erectile dysfunction following radical prostatectomy have decreased in the past few years due to nerve-sparing surgical techniques. However many months or even years after surgery still over 60% of the patients complain about erectile dysfunction [5-7]. Due to the diagnosis and treatment-related symptoms PCa patients also experience fundamental psychological and psychosocial issues. Both disease-specific (i.e. urinary, sexual and bowel function) and general health-related quality of life (i.e. energy/vitality and role performance) are negatively affected [8,9]. Over 50% of PCa patients that underwent a prostatectomy complain about distress and/or PCa-related anxiety prior to an inpatient rehabilitation measure [10]. Furthermore patients receiving irradiation are at a higher risk of developing depressive symptoms or fatigue [8,11]. As a consequence of hospitalization and the mentioned side effects, physical function and physical activity levels of most cancer patients decrease. Approximately 30% of the adult cancer survivors exercise less after diagnosis [12]; and only 39% of the PCa survivors meet the U.S. guidelines for physical activity [13].

Studies have shown that physical activities with PCa patients are feasible, safe, and efficient. They offer a multitude of possibilities to improve patients’ fitness, treatment- or disease-related side effects, well-being and quality of life during medical treatment, rehabilitation and in the aftercare [14,15]. Yet, despite the high incidence rate of PCa and the increasing evidence that physical activities are beneficial for PCa patients, there is a lack of supportive measures for male patients. In order to promote physical activity in cancer patients, effective strategies have to be explored, given that adopting and maintaining exercise is even more challenging for cancer patients than it is for healthy adults [16]. Since 1981, when the first official rehabilitative sports group for cancer patients was established at the German Sport University in Cologne, approximately 1000 groups were build up and provide a good care structure in Germany, however nearly 80-90% of the participants are breast cancer patients [17,18].

In order to improve the care structure for PCa patients and expand the current knowledge on the efficacy of physical exercise in PCa patients in the aftercare, rehabilitative sports groups in North Rhine-Westphalia (NRW) particularly for PCa patients were established and the effects of the offered supervised exercise program will now be evaluated. The outcomes of the study include aerobic endurance performance, physical activity levels, quality of life, incontinence, erectile dysfunction, relapse-relevant blood values, and adherence. The trial is characterized by its multicentric design, exceptionally long intervention period and holistic approach. To our knowledge this is the first study with a multi-modal exercise intervention that is conducted over 15 months, begins shortly after prostatectomy, when adverse effects are most pronounced, and examines the effects on incontinence and erectile dysfunction. We therefore hypothesize that long lasting physical, psychological, and psychosocial improvements can be achieved. Finally the involvement of different institutions enables a constant exchange and a seamless transition for patients from the hospital, to the rehabilitation centre, and finally to a rehabilitative sports group near their home towns.

## Methods/Design

For the following pilot project an interdisciplinary cooperation network consisting of hospitals, sports clubs, university institutions, the State Sport Association NRW, and the Cancer Society NRW was set up to establish rehabilitative sports groups particularly for PCa patients. In a second step a patient preference randomized controlled trial will be conducted in order to evaluate the effects of the multi-modal exercise program which will be offered to PCa patients in the newly founded rehabilitative sports groups. The study protocol has been approved by the ethics committee of the German Sport University Cologne and all patients will provide written informed consent prior to participation. While patients in the intervention group will exercise in a rehabilitative sports group for 15 months as described below (see Exercise intervention), patients in the control group will not take part in the exercise intervention.

### Subjects and sample size calculation

Patients are eligible to participate in the study if they have a malignant PCa disease and underwent a radical prostatectomy. Inclusion and exclusion criteria are listed in Table 1.

**Table 1:** Inclusion and exclusion criteria

**Inclusion criteria**	**Exclusion criteria**
- malignant PCa disease	- metastatic PCa disease
- primary treatment involved radical prostatectomy or combination therapy (radical prostatectomy + radiation)	- patients scheduled to receive hormone treatment or (neo-) adjuvant chemotherapy
- primary treatment is completed	- radiation as sole treatment
- primary treatment is at least 6 weeks, however no longer than 12 weeks ago	- severe cardiac disease (NYHA III-IV)
- written consent	- severe mental illness or chronic disease that rules out regular PA
	- alcohol, drug or medical abuse
	- insufficient German language skills
	- regular PA of more than 1 hour/week

*PCa*: prostate cancer, *PA*: physical activity, *NYHA*: New York Heart Association.

Based on studies by Windsor et al. and Crevenna et al. a moderate improvement in physical performance after a physical exercise intervention can be assumed when compared to a control group (effect size δ/σ=0.5) [19,20]. In order to detect an effect with a power of 80% and a two-tailed alpha of 0.05, 64 patients need to be observed in each group. Assuming a loss-to-follow-up of 30% across the entire intervention period, a total of 182 patients (2x64/0.7 = 182) will need to be observed.

### Recruitment

The medical assistant of the attending hospital will approach all potential study subjects and provide oral information about the intervention study. If a PCa patient complies with the inclusion criteria and signs a written consent approving the passing on of his contact details, the directing institution will call the patient after hospital discharge in order to provide further information. Those subjects entering the study will be invited to a preliminary conversation and for baseline measurements.

### Randomization

Based on a patient preference trial, patients that refuse randomization due to a strong group preference will receive the intervention of their choice while patients who give consent to randomization will be allocated randomly [21-23]. Randomization will be conducted by the Institute of Medical Statistics, Informatics and Epidemiology at the University of Cologne. It will be achieved based on a computer-generated list and sealed envelopes will then be passed on to the corresponding institution.

### Exercise intervention

After baseline measurements patients in the intervention group will exercise for 15 months in one of four rehabilitative sports groups located in a sports club closest to their home town. Physical exercise sessions will take place once a week for 60 minutes and will be financially covered by a patient’s statutory health insurance company. Additionally subjects will be asked to exercise another 60 minutes per week home-based or within the sports club. For the home-based exercise session the type of exercise is not predefined, however patients will be asked to include the exercises they learned and report the type and duration of their activity. All group trainers will be educated by the State Sport Association NRW and receive a semi-standardized exercise program particularly for PCa patients. The multi-modal exercise program mainly includes aerobic and strengthening exercises, as well as games and exercises that promote flexibility, coordination, relaxation skills, cognitive abilities, interaction, cooperation and communication. Trainers will choose the training content from a pool of exercises and the training intensities will be adjusted individually based on the patient’s condition and experience. Therefore new patients that join the group will slowly be introduced to the exercise program for the first few weeks. Since the intervention period is relatively long, the aim is to conduct a training that is motivational, diversified and fun. Components of the exercise program are described in Table 2. Patients in the control group will not participate in the intervention.

**Table 2:** Exercise pool of the semi-standardized multi-modal exercise program for prostate cancer patients

	**Content**	**N° of Exercises**	**Intensity**	**Aims**
**Endurance**	- Nordic walking	-	50-70% HRmax 11–15 RPE	Improvements in:
								-aerobic endurance
								-muscle strength
								-mobilization
								-wellbeing
**Stretching**	- stretching	16	2-3x 10–15 sec.	Improvements in:				
								-flexibility
**Strengthening**	- strengthening exercises incl. pelvic floor muscles	20	1. phase: 1–2 sets, 8–10 rep., 30% MVC	Improvements in:				
					- resistance exercises with equipment or strength training machines	28	2. phase: 2–3 sets, 8–15 rep., 30-50% MVC	-incontinence
								-muscle strength (esp. postural muscles)
								-flexibility
					-ADLs incl. pelvic floor muscles	16	3. phase: 3 sets, 15–20 rep., 50-60% MVC	-wellbeing
							4. phase: 3 sets, 12–15 rep., 70% MVC	
**Mixed games and exercises**	- relaxation exercises	8	Depending on exercise and patient’s condition	Improvements in:				
					- breathing exercises	5		
								-flexibility
					- partner exercises	6		-coordination
					- perception and coordination exercises	27		-relaxation skills
					- cooperative games	8		-cognitive abilities
								- interaction
								-cooperation
								-communication

*ADLs*: activities of daily living; *HRmax*: maximum heart rate; *MVC*: maximum voluntary contraction; *rep*.: repetitions; *RPE*: rating of perceived exertion.

### Measurements

Primary and secondary endpoints will be measured at three to five assessment time points in the intervention group (baseline/3 months after surgery, 6 months -, 9 months -, 12 months -, and 18 months after surgery/post-intervention) and twice in the control group (baseline/3 months after surgery and 18 months after surgery/post-assessment). Additionally the involved hospitals will assess two measures prior to surgery for all patients. Outcome measures, assessment methods and assessment time points are depicted in Table 3.

**Table 3:** Outcome measures, assessment methods, and assessment time points of the study

**Measures**	**Assessment methods**	**1. Test 1 Day Pre-OP**	**2. Test 6-12 Wk. Post-OP**	**3. Test 6 Mo. Post-OP (only IG)**	**4. Test 9 Mo. Post-OP (only IG)**	**5. Test 12. Mo. Post-OP (only IG)**	**6. Test 18. Mo. Post-OP**
				**15-month exercise intervention**			
Physical performance	Spiroergometry on treadmill		x		x		x
Incontinence	Pad-Test		x		x		x
PSA, Testosterone, Endostatin, VEGF	Blood Sample		x		x		x
Quality of Life	EORTC-QLQ-C30/PR 25	x	x	x	x	x	x
Erectile Dysfunction	IIEF – Questionnaire	x	x	x	x	x	x
Physical Activity Level (MET- Score)	Freiburger Questionnaire of Physical Activity		x	x	x	x	x
Compliance	Compliance Questionnaire		x	x	x	x	x

*EORTC-QLQ-C30/PR25*: European Organization for Research and Treatment of Cancer- Quality of Life Questionnaire- Cancer/Prostate Cancer Module, *IG*: intervention group, *IIEF*: International Index of Erectile Function, *MET*: metabolic equivalent, *Post-OP*: after surgery/primary treatment, *Pre-OP*: prior to surgery/primary treatment, *PSA*: prostate-specific antigen, *VEGF*: vascular endothelial growth factor.

#### Primary and secondary endpoints

##### Physical fitness and physical activity levels

In order to assess the primary endpoint, aerobic endurance, patients will perform a spiroergometry with a submaximal test protocol, which was created by a team of sport scientists and physicians. The new protocol is based on the Hollmann/Venrath protocol and was developed in order to enable a spiroergometry with PCa patients shortly after radical prostatectomy [24,25]. Instead of using a cycle ergometer we will use a treadmill to spare the surgical area. The initial speed of 0.8 m/s will be increased moderately by 0.2 m/s every 3 minutes until one of the following conditions occur: the minute ventilation or ventilation equivalent increases excessively, the ventilation equivalent reaches a value between 28 and 30, the metabolic respiratory quotient exceeds 1.0, muscular fatigue sets in, the patient feels exhausted (borg scale: ≥ 17) or any complications interfere with the testing procedure. The aim is to reach lactate values of  > 2.0 mmol/l blood by the end of the test, since this will be the primary endpoint of aerobic endurance performance testing. The research assistant that conducts the tests is a sport scientist who will not be involved in the training sessions and will be uninformed about the group allocation. Physical activity levels will be evaluated with the Freiburger Questionnaire of Physical Activity, a standardized questionnaire that retrospectively assesses the physical activities performed by a patient during the last 4 weeks. Based on the patients answers MET-Scores will be calculated [26,27].

##### Incontinence

In order to examine the effects of the exercise program on urinary incontinence patients will perform a 20-Min Pad-Test. The Pad-Test is a standardized test that objectively measures urine loss, as patients weigh their pads before and after conducting a set of incontinence-induced activities [28,29].

##### Erectile dysfunction

Since erectile dysfunction is a common problem following radical prostatectomy and the influence of physical activities on the erectile function of PCa patients is mostly unknown [30], the International Index of Erectile Function will be included as an assessment method for erectile dysfunction. The questionnaire comprises 15 questions that determine the effects that a patients erection problems have had on his sex life over the past four weeks [31,32].

##### Blood values

In order to screen the dynamic of the carcinogenic disease, prostate-specific antigen (PSA), total testosterone, endostatin, and VEGF values will be assessed by taking blood samples. All four blood values influence the tumor environment and may be altered by physical activity [33-35].

##### Psychological and psychosocial assessments

To assess quality of life, patients will complete a questionnaire that was developed by the European Organization for the Research and Treatment of Cancer and has been well proven in its validity and reliability [36]. Besides the EORTC-QLQ-C30 questionnaire, a 30-item scale, the PCa specific module EORTC-QLQ-PR25 with another 25 questions will be attached [37]. The assessed domains include physical function, emotional state, social interaction, global health status/quality of life, and several symptom scales.

##### Adherence

Patient’s adherence concerning medical attendance, the physical exercise program, and further measures that are beneficial to health (e.g. cancer screening, balanced nutrition) will be assessed based on an adherence questionnaire, which has successfully been used in a study with schizophrenic patients [38], since an adequate adherence questionnaire for cancer patients in German language could not be found. The modified tool includes nine questions which can be answered on a scale of 1 (very good) to 6 (very bad). The number of exercise sessions attended will be recorded by the group trainers.

##### Anthropometric assessments

At baseline and final post-assessment testing height and weight will be measured in order to calculate the patients’ body mass index.

## Discussion

The survival rate of PCa patients has continuously increased in the last few years. This development has generated interest in behavior and lifestyle interventions that might further improve disease outcomes and quality of life in PCa survivors. Latest investigations show that resistance and aerobic training may improve physical fitness, fatigue, quality of life, and body composition in PCa patients [39]. These positive effects are now underlined by an observational finding that suggests that post-diagnosis physical activity is even associated with improved survival rates in PCa patients [40].

Regardless of these positive observations and the increasing evidence in the field of physical activity in PCa patients, a majority of patients are inactive, physicians hesitate to prescribe physical exercise, insurance coverage is insufficient, lifestyle and behavior interventions lack prioritization in the health care systems and patients are uninformed [16]. In order to improve the current situation and promote physical activity in PCa patients effective strategies have to be identified, including further research in the field, active involvement and briefing of oncologists, exercise therapists and insurance companies and tailored exercise programs [16].

The ProRehab project aims to create a unique and lasting exercise offer for PCa patients and improve the knowledge in the field of physical activity and PCa. To date the first official and certified rehabilitative sports groups particularly for PCa patients have been established in Germany. This preliminary success is based on a close cooperation between oncologists/urologists, sports clubs, university institutions, the Cancer Society NRW, the State Sport Association NRW and the statutory health insurance companies. By combining science, practice, and public relations a great contribution to the care structure for PCa patients could already be made.

We planned the described patient preference randomized controlled trial to assess the efficacy of the offered rehabilitative sports program on physical activity levels, physical fitness, incontinence, erectile dysfunction, blood values, and quality of life. According to Keogh and MacLeod (2012) group-based physical activities offer greater benefits for prostate cancer patients than home-based interventions and aerobic training should best be supplemented by resistance exercises [41]. While the positive effects of pelvic floor muscle exercises on urinary incontinence have been well proven [42], Wolin et al. now found that physically active PCa patients in general have lower incontinence rates [43]. The influence of physical activity on sexual functioning in PCa patients has hardly been studied [30]. We are therefore, for possibly the first time, examining the effects of a multi-modal exercise program on incontinence and erectile dysfunction in PCa patients.

Depending on the findings of our study and future investigations the exercise program offered in the rehabilitative sports groups needs to be adjusted, since evidence based guidelines for physical activities with PCa patients have not yet been published. Especially randomized controlled studies with longer intervention periods (>1 year) or follow-up measurements that examine the long-term effects of physical activities in PCa patients are still missing. To our knowledge this is the first study in which PCa patients exercise in a supervised setting for 15 months. By offering a physical exercise intervention that is group-based, lasts for 15 months and involves a motivational and multi-modal exercise program we are convinced that a long-term lifestyle change in PCa patients can be promoted.

## Abbreviations

PCa, Prostate cancer; NRW, North Rhine-Westphalia; EORTC-QLQ-C30, European organization for the research and treatment of cancer- quality of life questionnaire-cancer; EORTC-QLQ-PR25, European organization for the research and treatment of cancer- quality of life questionnaire- prostate cancer; MET, Metabolic equivalent; VEGF, Vascular endothelial growth factor; IG, Intervention group; IIEF, International index of erectile function; Post-OP, After surgery; Pre-OP, Prior to surgery; PSA, Prostate-specific antigen; NYHA, New York heart association.

## Competing interests

The authors declare that they have no competing interests.

## Authors’ contributions

FTB initiated the project and completed the study protocol; FTB, WB, MB, SM, JZ and EMZ developed the concept of the project and FTB and WB will direct the study; MB SM and JZ will provide access to patients; EMZ and FTB will implement the protocol, acquire and analyze data; all authors read and approved the final manuscript.

## Pre-publication history

The pre-publication history for this paper can be accessed here:

http://www.biomedcentral.com/1471-2407/12/312/prepub
